# Nanoarchitectonics-based electrochemical aptasensors for highly efficient exosome detection

**DOI:** 10.1080/14686996.2024.2345041

**Published:** 2024-04-22

**Authors:** Aisha Javed, Na Kong, Motilal Mathesh, Wei Duan, Wenrong Yang

**Affiliations:** a School of Life and Environmental Science, Centre for Sustainable Bioproducts, Deakin University, Geelong, VIC, Australia; b School of Medicine, Faculty of Health, Deakin University, Geelong, VIC, Australia

**Keywords:** Exosomes, biomarkers, aptamer, nanoarchitectonics, electrochemical detection

## Abstract

Exosomes, a type of extracellular vesicles, have attracted considerable attention due to their ability to provide valuable insights into the pathophysiological microenvironment of the cells from which they originate. This characteristic implicates their potential use as diagnostic disease biomarkers clinically, including cancer, infectious diseases, neurodegenerative disorders, and cardiovascular diseases. Aptasensors, which are electrochemical aptamers based biosensing devices, have emerged as a new class of powerful detection technology to conventional methods like ELISA and Western analysis, primarily because of their capability for high-performance bioanalysis. This review covers the current research landscape on the detection of exosomes utilizing nanoarchitectonics strategy for the development of electrochemical aptasensors. Strategies involving signal amplification and biofouling prevention are discussed, with an emphasis on nanoarchitectonics-based bio-interfaces, showcasing their potential to enhance sensitivity and selectivity through optimal conduction and mass transport properties. The ongoing challenges to broaden the clinical applications of these biosensors are also highlighted.

## IMPACT STATEMENT

This review emphasizes the significant impact of integrating nanoarchitectonics into aptamer-based electrochemical biosensors for exosome detection, thereby enhancing early disease detection and monitoring disease progression in clinical settings.

## GRAPHICAL ABSTRACT

**Figure:**
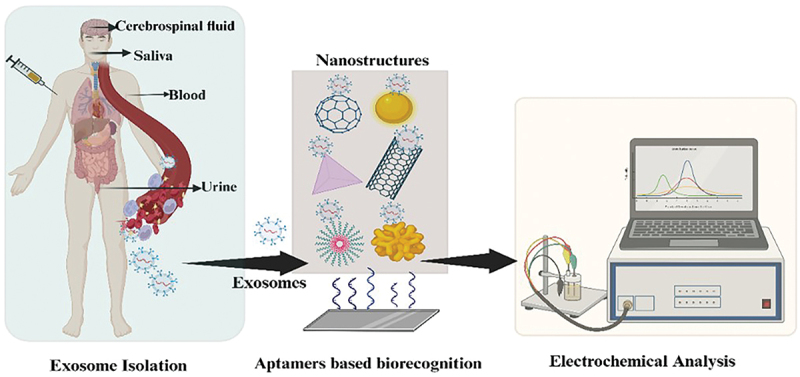

## 1. Introduction

Exosomes, membrane-enclosed spherical or oval-shaped nanometric subtypes (30 to 150 nm) in extracellular vesicles (EVs) family [1,2], play a pivotal role in cell-to-cell communication, regulating cellular metabolism, promoting adaptation and survival in living organisms [3]. Derived from various cell types, including neurons, tumors, immune cells, and stem cells [4–7], exosomes exhibit immense stability and widespread existence in body fluids such as cerebrospinal fluid, tears, urine, blood, saliva, breast milk and amniotic fluid [8,9]. They accumulate and circulate freely, carrying a variety of biological molecules from parent cells to nearby or distant recipients, potentially crossing the blood – brain barrier [10,11]. Initially, exosomes were disparaged as a ‘cell dust’ and considered as a machinery to dispose of cellular constituents, until the discovery of certain nucleic acid molecules (messenger RNA-mRNA, micro RNA-miRNA) in the exosome structure that cemented their key cell-derived messenger status of these nanoscale vesicles [12,13]. This revelation led to growing interest in research towards exosomes-based disease biomarkers, particularly micro RNAs (miRNA), for instance miRNA-1, miRNA −21, miRNA-141, miRNA-146, miRNA-181 and miRNA-210 [14,15]. Traditional biomarkers such as proteins and RNA isolated from diverse body fluids for disease detection, exhibit limitations such as low abundance, high heterogeneity (unclear molecule of origin), low accuracy, reproducibility, and specificity [16]. In contrast, exosomes offer advantages in terms of heterogeneity and stability in various fluids, addressing limitations in abundance, accuracy, and reproducibility. Exosomes emerge as non-invasive molecular markers for early detection of many malicious diseases like cancers, neurodegenerative disorders, cardiovascular diseases, pregnancy abnormalities, immunological responses, and infectious diseases [17–19]. Consequently, significant emphasis is placed on developing credible and sensitive techniques for detecting exosomes and their surface biomarkers to enhance clinical prognosis [6].

Effective exosome analysis relies on two factors: enhanced exosome capture and optimized performance of detection techniques. Various approaches for exosome isolation have been reported, including density gradient centrifugation, ultra-centrifugation, size exclusion chromatography, polymer precipitation, and immune affinity capture [20]. Additionally, diverse analytical techniques have been developed for the quantitative detection of exosome biomarkers, Western analysis, enzyme linked immunosorbent assay (ELISA), flow cytometry, nanoparticle tracking analysis (NTA), transmission electron microscopy (TEM), mass spectrometry, microarray, next-generation sequencing, and quantitative reverse transcription polymerase-chain reaction (qRT-PCR) [5,21–23]. Despite their efficacy and robustness, these techniques still suffer from several disadvantages due to the complexity of analysis, expensive sample pretreatment owing to matrix interference, and time-consuming operations. Therefore, they are routinely used for point of care testing of exosomes in clinical samples.

To overcome these challenges, electrochemical biosensors like glucose meters show incredible potential for routine disease screening and surveillance in whole blood and other body fluids. In 2022, the global market of biosensors was estimated to be 24.9 billion US dollars, with an anticipated 8% compound annual growth rate from 2022 to 2030. Despite this progress, most biosensors are restricted to academic contexts rather than demonstration of their viability at commercial scale [24]. Electrochemical biosensors also face sensitivity and specificity issues due to electrode surface inactivation and biofouling, especially with very low concentration of targeted biomarkers (pg-ng mL^−1^) in complex biological samples [25]. The major reason underlying this limitation is the non-discriminatory binding of molecules on the electrode surface that impedes electron transfer and consequently results in low current and sensitivity. Unlike glucose sensor, where semi-permeable membranes prevent large biomolecules from approaching to the electrode surface, clinically relevant biomarkers, being of similar size to proteins, cannot benefit from the semi-permeable membranes. Also, in some diseases where the highly complex matrix of serum or blood contains extremely low concentrations of biomarkers, detection of the disease markers is very difficult. Optimal specificity, sensitivity and selectivity of electrochemical biosensors, therefore, relies greatly on the choice of affinity ligands employed in the sensor. Biorecognition probes (antibodies, enzymes, and nucleic acids) need enhanced binding affinity for their targets but are limited in specificity and selectivity due to their challenging physical and chemical modification [26]. While antibodies (or monoclonal antibodies) have achieved impressive success, they suffer from limitations such as high production costs, batch-to-batch variation, and laborious purification methods used during the production. A significant drawback is their limited surface area to bind small target molecules, also the binding affinity does not reach the sub-nM level [27]. Therefore, aptamers are considered an efficient alternative to address the challenges of antibodies. The key diagnostic characteristic of aptamer-based detection approaches are their remarkable selectivity, sensitivity and specificity. Aptamers, with a *K*_*D*_ as low as 1 × 10^−12^ M [28], have been found to overcome these problems as they holds the potential to detect extremely low concentrations of target molecules with high accuracy [29]. The recent application of a nano-bioengineered signal amplification platform in electrochemical biosensors has opened a new frontier for ultrasensitive detection of molecular markers using conducting nanomaterials. Nanobiosensors based on nanostructured materials serve as potential devices for enhanced detection. Nanomaterials exhibit extremely small diameters, distinct electrocatalytic characteristics, effortless surface tuning and extensive surface area that allow high-affinity adsorption of bio receptors, preventing non-discriminatory binding [30]. These nano-engineered biosensors facilitate the analysis of even minute amount of samples (from pL to nL) in a limited time span (minutes and seconds), thereby enhancing the accuracy of diagnostic tests [31].

## 2. Exosomes in diagnostics

### 2.1. Exosome biogenesis and release

**Figure 1.**
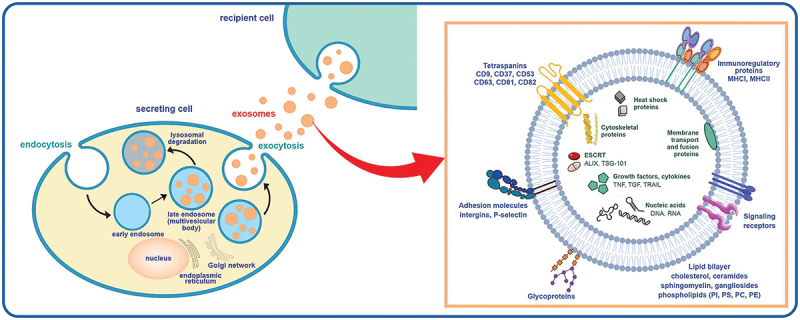
Exosome biogenesis and its molecular components. Reproduced by permission from [32], copyright [2022, American Chemical Society].

Exosomes biogenesis follows a three-step constitutive process originating from the endosomal system. As shown in Figure 1, the process involves the uptake of intracellular fluids, leading to forming a tubular early endosome that enclose cellular genetic material (mRNA, DNA, and non-coding RNA) and proteins present of the cell in the cytoplasm. Following that, early endosomes mature into late endosomes with changes in endosomal content and membrane composition. By engulfing the cytosolic proteins (peripheral and transmembrane), these mature endosomes form intraluminal vesicles (ILVs) [33]. Finally, these spherical mature endosomes also called multivesicular bodies (MVBs) either fuse with the lysosomes and get degraded, or fuse with plasma membrane to release ILVs (now called as exosomes) into the outer environment [34,35]. The intracellular machinery associated with exosomes synthesis, cargo sorting and release includes different factors operating through the endosomal sorting complex required for transport (ESCRT) dependent pathway via engaging specific signal molecules and the ESCRT independent pathway. The ESCRT is a four-protein complex (ESCRT-0,1,2,3) associated with other proteins like ALIX, VPS4, Tsg101 [34,36]. ESCRT-0 assembles ubiquitinated peptides into ILVs, while ESCRT-1 and 2 aid in cargo internalization. ESCRT and VPS4 collaboratively detach the ESCRT complex from ILVs in the next step [37]. The ESCRT dependent pathway coordinates with ESCRT independent pathway. The later involves lipid rafts contains sphingolipids, glycosyl-phosphatidylinositol and specific membrane proteins such as tetraspanins, contributing to genomic material sequestration and exosome formation [38]. This process organises endosomal membranes into distinct domains, called as tetraspanins-enriched membrane domains (TEMs), essential for vesicular fission or fusion. Moreover, TEMs also appoint prospective ligands to facilitate receptor mediated uptake of exosomes by target cells. Multiple tetraspanin candidates like CD9, CD63 and CD81 concentrate on exosomal surfaces, and act as marker proteins for vesicle capture [39].

The fusion of mature endosomes with the plasma membrane triggers various biochemical reactions including GTPases (like Rab27a and b), motor proteins (for example, dynein and kinesin) together with skeletal proteins responsible for the release of exosomes intro extracellular matrix (ECM). Proteins related to the SNARE family (v-SNARE, t-SNARE) play a crucial role in providing mechanical force to promote fusion of late endosomes with plasma membranes for exosomes release [40]. In ECM, upon release into biofluids, exosomes transfer their cargo to target cells through three processes: proximity interaction with cell membrane receptors, immediate attachment to cell membrane and ingestion by recipient cells [41]. Notablly, the direct budding of exosomes from the plasma membrane without the involvement of the endocytic pathway has also been reported [42].

### 2.2. Molecular structure of exosomes

Owing to the stable composition of protein and nucleic acids, exosomes function as carriers of vital biomarkers originated from parent cells and have attracted increasing attention in disease detection and targeted drug delivery [19]. As shown in Figure 1, the exosomal lipid bilayer is primarily composed of phospholipids [43], forming a continuous barrier to intracellular components including DNA, mRNA, miRNA, cirRNA, heat shock proteins, growth factor receptors, enzymes, biogenesis proteins, lipid-related proteins and phospholipases. These constituents function as molecular messages, orchestrating communication between parent and recipient cells [44–48]. The extracellular assay in the lipid bilayer, including phosphoglycerides with long and saturated fatty-acyl chains, cholesterol, and sphingolipids, ceramides [49], saccharide chains, lactadherin and membrane transport and fusion proteins, serve as signals for specific cell receptors, facilitating intricate cellular communication between target cells and exosomes [50]. Initially discovered in 1981 as vesicles eliminating obsolete proteins [51,52], their primary role is now recognised as transport carriers actively participating in cell–cell communication, providing insights into intracellular and extracellular biological molecules [53]. For those seeking in-depth and current information on exosomes, resources like Vesiclepedia and ExoCarta provide up-to-date and comprehensive insights [54].

### 2.3. The role of exosomes biomarkers in diagnostics

#### 2.3.1. General biomarkers

For decades, early diseases diagnosis has been a main goal in medical diagnostics, providing a great opportunity for timely treatment. Biomarkers play a crucial role in this pursuit, offering objective insights into pathogenic and normal biological processes [55]. Various body fluids, such as plasma, serum, cerebrospinal fluid, amniotic fluids, and saliva, have been extensively studied for biomarker detection and validation [56]. Serum and plasma samples stand out as ideal mediums for identifying biomarkers in clinical systems due to their rich composition of lipids, proteins, products from different body tissues, and metabolites. However, the challenge arises from the excessive concentration of diverse proteins in the blood proteome, impeding the accurate detection of low-abundance protein biomarkers [57]. To address this limitation, innovative techniques and technologies, as highlighted in recent studies [58–61], aim to enhance the detection of low-abundance proteins in various bodily fluids. These advancements pave the way for more accurate and reliable early disease diagnosis.

#### 2.3.2. Exosome biomarkers

Detecting exosomes-based biomarkers has a distinct advantage in significantly reducing sample complexity compared to analyzing entire biological fluids. The consistent and stable membrane architecture of exosomes in bodily fluids makes them promising non-invasive biomarkers for disease diagnostics [62]. Recent research has predominantly focused on detecting exosome biomarkers in plasma or serum, which is riched in disease-associated DNA, proteins, and RNA. Blood samples, obtained through minimally invasive techniques, provide generous amounts of exosomes secreted by diseased cells, surpassing those from normal cells [63]. Current diagnostic exosome research targets cardiovascular diseases, cancer, infectious diseases, and central nervous system disorders. Comprehensive lists of exosome biomarkers for these conditions are available in Table 1, highlighting the potential for early detection even before symptoms appear. Exosomes emerge as a promising source of biomarkers in the early stages of diseases, holding tremendous potential in advancing diagnostic capabilities [108].

**Table 1.** Exosomal biomarkers in the diagnosis of different diseases.

Diseases		Exosome Biomarkers	Ref.
Cancer	Glioblastoma	Human epidermal growth factor receptor (EGFR) and EGFR variant (v) III mutation (EGFRvIII), CD63, and epidermal growth factor receptor variant-III	[64,65]
	Breast Cancer	CD24, CD63, and EGFR, miR-1246, CD63 aptamer and EpCAM aptamer, miR-210, 2 exosome markers; CD9, CD63, 4 cancer markers; CD24, CD44, EpCAM, and the human epidermal Growth factor receptor 2 (HER2), CD44, miR-21, miR-27a and miR-375, CD47	[66–73]
	Ovarian Cancer	CA-125, EpCAM, CD24, claudin proteins, miR-21, miR-141, miR-200a, miR-200c, miR-200b, miR-203, miR-205, and miR-214 EpCAM-phosphatidylserine (PS)-positive exosomes	[74–76]
	Prostate Cancer	PSA and PSMA, miR-21, miR-574-3p, EpCAM, and epidermal growth factor receptor (EGFR), miR-17, miR-18a, miR-20a, miR-93, miR-106b and thelet-7family members, PCA-3 and TMPRSS2:ERGmiR-1290 and miR-375	[77–81]
	Colorectal Cancer	A33, EpCAM, CD34	[82]
	Lung Cancer	EGFR, CEA, CYFRA 21–1, ENO1, NSE, CA 19–9, CA 125 and VEGF, miRNA-21	[83]
Infectious Diseases	Tuberculosis	Up regulation of Ag85 A,HspX, K85R, HSP-90, HSP60, HSP70, Antigen-85C, Apa, bfrb, glcB, HspX, katG, MPT64, Mannosylated Lipoarabinomannan, lipoarabiomannan	[84–87]
	HIV	Upregulation of TAT protein, ISGs, ISG15, ISG56, A3G	[88–91]
	HBV	Up regulation of HBsAg, HBeAg, HBsAb, HBV-DNA, IFN-α	[92,93]
	HCV	Up regulation of HCV virion, MiR-122.	[94–96]
	Epstein Barr virus (EBV)	Up regulation of EB particles, proteins, RNAs, LMP-1, Galectin-9, miR-BART15-3p	[97,98]
Cardiovascular Diseases	Acute Myocardial Infarction	hsa-miR-1180-3p, hsa-miR-3615, hsa-let-7i-5p, hsa-miR-106b-5p, hsa-miR −143-3p, hsa-miR-17-5p and hsa-miR-1273 h-3p; 1 miR-126, miR-21 PTEN, lncRNA: ENST00000556899.1 and ENST00000575985.	[99,100]
	Unstable Angina Pectoris	miR-126, miR-21 and PTEN	[100]
Neurodegenerative diseases	Alzheimer’s Disease	Aβ-oligomer, p-tau, miR-135a, BACE1-AS, miR-34b, miR-125b and miR −130b, GAP43, neurogranin, SNAP25, and synaptotagmin 1, hemoglobin	[101–105]
	Parkinsons Disease	α-Synuclein and clusterin, STX-1A and VAMP-2, let-7d, miR-15b, miR-24, miR-142-3p, miR-181c, and miR-222, miR-153 and miR-223.	[106,107]

## 3. Exosomes detection and analysis

### 3.1. The limitations of conventional techniques

Detecting exosomes involves complex steps, including pre-isolation and molecular physicochemical and quantitative characterization, relying on analytical methodologies pre-isolation [109]. Current assays like ELISA and Western analysis require large sample volumes and involve intricate workflows, hindering efficiency [110]. Conventional flow cytometry, although a high-throughput analysis technique, is limited in screening exosomes with a diameter less than 500 nm since it relies on light scattering after introducing exosomes in the laminar fluid. Novel screening techniques, such as integrated microfluidics, fluorescence assay, surface enhanced Raman spectroscopy, colorimetric detection, surface plasmon resonance [111–114], have shown increased sensitivity, lower exosomal detection cost and simplified process in real-time screening. However, many require expensive pre-treatment and involve multistep processes, challenging accurate exosome identification. The immense low concentration of exosomes from tumor cells in early-stage tumors poses a hurdle, especially with human blood plasma containing 10^10^ particles/mL. This leads to the utilization of biosensor technology that allows for quantification of analytical data in-mixture. Electrochemical biosensing technology is one of the most commercially effective biosensing technology for the real-time on-site diagnostics for clinical diseases [115].

Aptamer-based biosensors have emerged as indispensable tools in detection technologies, particularly in analysis of exosomes owing to their heightened sensitivity, rapid response times, minimal sample volume requirements, and portability. Over the past few years, six main types of aptamer-based biosensors have been reported for exosome detection, encompassing electrochemical, colorimetric, fluorescent, surface plasmon resonance (SPR), surface-enhanced Raman scattering (SERS), and dual-mode sensors. Among these, electrochemical biosensors have gained considerable interest due to their enhanced sensitivity, adaptability to novel methodologies, rapid response durations, and potential for miniaturization, coupled with cost-efficient operation [116]. Notably, electrochemical detection does not hinge on sample volume; even minute samples can be utilized for measurements, facilitating achievement of very low detection limits with minimal or no sample preparations. Electrochemical experiments can be conducted on turbid or colored samples like whole blood, unhindered by interfering factors such as red blood cells, fat globules, bilirubin, or hemoglobin [117].

### 3.2. Aptamer-based electrochemical detection of exosomes

Despite their relatively recent introduction, aptamer-based electrochemical biosensors (also known as aptasensors) have been extensively explored as diagnostic tools for exosome detection among different techniques [118,119]. Integrated systems are easier to be fabricated over other current molecular techniques, which advance their application in point of care testing. The design of electrochemical biosensors is critical in the identifying diseases through specific molecular markers. These biosensors incorporate either antibodies or nucleic acids, particularly aptamers to achieve sensitive, rapid, and selective detection of exosomes. Table 2 provides a comparative analysis of aptamer and antibody-based electrochemical biosensors, demonstrating that aptasensors exhibit higher sensitivity in detecting exosomes compared to antibody-based counterparts. Antibodies are gradually being replaced due to drawbacks in high cost of manufacturing, batch to batch variations and short shelf life [130]. In contrast, aptamers, synthetic nucleic acid with 25–80 nucleotides in length, provide a durable, cost-effective, and highly stable alternative [131]. Often known as ‘chemical antibodies’, aptamers exhibit enhanced affinity and specificity for their targets, functioning effectively even in non-physiological conditions, where regular antibodies may falter [119,132]. Moreover, aptamers isolation is achieved through an in vitro reiterative approach known as systemic evolution of ligands by exponential enrichment (SELEX) [133,134]. To expedite selection and enhance the hit rate, more adaptable SELEX approaches such as capillary electrophoresis SELEX, counter SELEX, in vivo SELEX, microfluidic SELEX, and high-throughput SELEX have been introduced which can be applied to complicated targets (e.g. whole cells, animals, bacteria or viruses) [135]. Chemically synthesized aptamers can be easily modified to enhance their lifespan and stability in the bloodstream or to immobilize and target them to specific locations [136].

**Table 2.** Comparison of recent antibody and aptamer-based electrochemical biosensors for detection of exosomes.

Biomarkers	Biorecognition Probe	Signal Amplification Techniques	Disease	Real Samples	Dynamic Range (exo/mL)	Detection Limit (exo/mL)	Ref.
CD63	Antibody	None	Not specified	Spiked serum	Not specified	6.71 × 10^7^	[120]
CD9	Antibody	AuNPs@MCF/MWCNTs ternary nanocomposite	Breast cancer	Spiked plasma	10^5^–10^10^	7 × 10^4^	[121]
CD9,CD63, CD81, CD24, CD44, CD54, CD326, CD340	Antibody	None	Breast cancer	Serum	Not specified	<10^5^	[122]
CA-125 and CD9	Antibody	None	Cancer	No	Not specified	7.1 × 10^8^	[123]
CD9,CD63, CD81, CD326, ALP	Antibody	None	Breast cancer	Serum	Not specified	10^8^	[124]
EGFR,CD9 CD63	Affibody	None	Non-small cell lung cancer	No	Not Specified	0.8 × 10^9^-3.5 × 10^9^	[64]
EGFR	Peptide	Metal organic frameworks (MOFs)	Glioblastoma	Serum	9.5 × 10^6^-1.9 × 10^10^	7.83 × 10^6^	[125]
EpCAM and CD63	Aptamer	DNA walker, Ag@C nanocomposites	Breast cancer	30–60% fetal bovine serum	10^5^-75 × 10^6^	4 × 10^4^	[126]
CD63	Aptamer	SiO_2_@Ag	Breast cancer	Serum	4.2 × 10^5^ −4.2 × 10^11^	1.08 × 10^6^	[127]
CD63	Aptamer	Multipedal DNA walker strategy	Cervix cancer	No	10^4^ −2 × 10^6^	6 × 10^3^	[128]
CD63	Aptamer	MOFs, dual signal output	Breast cancer	Plasma	1.3 × 10^2^ to 2.6 × 10^5^	100	[127]
CD63	Capture probe-free, aptamer for recognition	MOFs, (Hybridization chain reaction) HCR, DNAzymes	Breast cancer	10% fetal bovine serum	1.7 × 10^4^ to 3.4 × 10^8^	5 × 10^3^	[129]

Given the ability of aptamers to bind to small molecules and protein ligands [136], aptamers are immobilized as promising biological recognition molecule for the detection of exosome-linked membrane proteins like tetraspanins (CD63, CD9, CD81), membrane transport and fusion proteins (flotillin, Annexins, RAB GTPase lineage), proteins involved in the biogenesis of multi vesicular body (e.g TSG101, Alix) and lipid attached extracellular proteins. Additionally, certain transmembrane protein receptors along with adhesion proteins (like HER2, EpCAM, EGFR) are also used as exosome disease biomarkers. Recently reported aptamers sequences specific for various exosome biomarkers are listed in Table 3. Aptamers against such proteins exhibit selectivity and are widely used in diseases diagnosis [156]. The remarkable efficiency of aptamers in binding to their targets can be attributed to a combination of geometrical complementarity via internal base-pairing, resulting from diverse structural features such as stems, bulges, loops, pseudoknots, triplex or G-quadruplex structures, hairpins, and molecular connections. Intriguingly, these structural features of aptamers arise from non-covalent and intra-molecular interactions, including Van der Waal forces, electrostatic attraction, pi-stacking in aromatic rings, and hydrogen bonds [157].

**Table 3.** Summary of recently reported aptamers that can bind to exosome biomarkers.

Exosome biomarker	Aptamer Sequences (5′-3′)	Ref.
CD63	CACCCCACCTCGCTCCCGTGACACTAATGCTA	[137]
	GTGGGGTGGACGAGGGCACGTGATTACGTA	[138]
	TAACACGACAGACGTTCGGAGGTCGAACCCTGACAGCGTGGGC	[139]
EpCAM	CACTACAGAGGTTGCGTCTGTCCCACGTTGTCATGGGGGGTTGGCCTG	[2]
	EpCAM-1: CACTACAGAGGTTGCGTCTGT	[140]
	EpCAM-2: CCCACGTTGTCATGGGGGGTTGGCCTG	
HER2	TACCAGTGCGATGCTCAGTGCCGTTTCTTCTCTTCGCTTTTTTTGCTTTTGAGCATGCTGACGCATTCGGTTGAC	[141]
	GGGCCGTCGAACACGAGCATGGTGCGTGGACCTAGGATGACCTGAGTACTGTCC	[142]
CEA	TCGCGCGAGTCGTCTGGGGAACCATCGAGTTACACCGACCTTCTATGTGCGGCCCCCCGCATCGTCCTCCC	[143]
MUC1	TACTGCATGCACACCACTTCAACTA	[144]
	TTGATCCTTTGGATACC	[145]
	TCCGAGTTTCCCTGCCCCAACCTCCACCTGGGGTCAATAA	[146] [147]
AFP	AACAAGCTTGGCGGCGGGAAGGTGTTTAAATTCCCGGGTCTGCGTGGTCTGTGGTGCTG	[148] [149]
	GGCAGGAAGACAAACAAGCTTGGCGGCGGGAAGGTGTTTAAATTCCCGGGTCTGCGTGGTCTGTGGTGCTGT	[150]
Nucleolin	GGTGGTGGTGGTTGTGGTGGTGGTGG	[151]
PTK7	ATCTAACTGCTGCGCCGCCGGGAAAATACTGTACGGTTAGA	[152]
PSMA	GCGTTTTCGCTTTTGCGTTTTGGGTCATCTGCTTACGATAGCAATGCT	[153]
PSA	GGGCGGGGCGGACGAGACAGTAAGGGCTGTGGGTGTGGTG	[154]
PRFI, Fasl	TCGGTCGGCTCAGTTGAGGTTTAACCCAGTAGGCGCACCA	[155]

The primary challenge associated with aptasensors is their susceptibility to degradation by nucleases, thereby compromising analytical performance in real matrices due to the presence of nucleases in biological fluids. Moreover, the low concentration of disease-related exosomes in body fluids and the adsorption of nonspecific proteins from blood samples onto electrodes lead to electrode fouling, hindering charge transfer and consequently deteriorating the performance of electrochemical biosensors [156,158,159]. To address these challenges, protective structures and surface modifications for aptamers are necessary to ensure the stability of biorecognition probes in complex media. Nanoarchitectonics is being employed in electrochemical biosensors to modify electrode surfaces, integrating nano-bio-electrochemical systems that offer novel and distinct properties due to the intrinsic characteristics of nanostructures on the electrode interface, thereby amplifying biosensor sensitivity [159]. The utilization of novel nanostructures alongside aptamer-based amplification techniques significantly enhances detection sensitivity while also ensuring aptamer stability [156]. Aptamers can be conjugated with various types of nanostructures to serve as ligands. When attaching aptamers to nanoparticles, the surface charge plays a crucial role. Direct attachment of aptamers on cation-rich surfaces, such as polyethylenimine, may lead to the formation of a complex between the aptamer and polyethylenimine, hindering the proper folding of aptamers onto target molecules and rendering them ineffective. Conversely, aptamers can be directly immobilized on nanoparticles composed of neutral materials. For example, polymers like polylactic-co-glycolic acid (PLGA) or poly-lactic acid (PLA) are suitable for conjugation with aptamers. Therefore, the binding affinity and selectivity exhibited by aptamers in solution are crucial for aptamer immobilization. This is typically achieved through two strategies: covalent coupling of aptamers to linkers attached to the surface or, in certain cases, non-covalent conjugation [160]. In non-covalent methods, interactions such as hydrogen bonding, electrostatic forces, van der Waals forces, and pi–pi interactions facilitate bond formation between nanomaterials and aptamers [161]. DNA nucleobases utilize hydrogen bonds and pi–pi interactions to conjugate with graphene oxide [158]. Efficient modifications have been developed based on these interaction methods. For example, imidazolium ring structures or biotin–avidin interactions are introduced to provide self-assembly characteristics [162]. Covalent conjugation methods are more frequently used in aptamer-nanomaterial conjugation due to their stability compared to non-covalent approaches. These interactions are widely employed for synthesizing nanomaterials with various functional groups [58]. Additionally, scientists use linkers to attach aptamers via covalent interactions [163]. For instance, in the case of gold nanoparticles, thiol is attached as a linker through thiol-maleimide chemistry [164]. Carboxylic acid, amine, and hydroxyl groups are also present on the surfaces of certain nanomaterials. Aptamers are conjugated through carbodiimide chemistry [165]. For functionalization, amine groups can react with N-hydroxy-succinamide esters or isothiocyanate. Nanomaterials with hydroxyl groups on their surfaces form a reactive intermediate via carbonyldiimidazole. Epoxy groups can also be employed for functionalization, interacting with aptamers with amine groups [166].

## 4. Nanoarchitectonics based electrochemical biosensing for improved detection efficiency

The strategic use of nanoarchitectonics has become crucial in the fast-evolving field of electrochemical biosensor research. Nanoarchitectonics is a multidisciplinary field of research and technology that involves designing and manipulating materials at the nanoscale to create new structures and functions [167]. Nanoscale precision in device fabrication and sensing material composition is required for advancements in biosensor performance, including selectivity, sensitivity, and usability. Nanoarchitectonics, which derives from nanotechnology in device fabrication and molecular self-assembly for sensing materials, enables both sensing material composition and nanoscale precision in device construction [168]. To build superior biosensor platforms, this paradigm shift needs the synergistic incorporation of nanocomposites and nanohybrids [169–171]. The incorporation of nanomaterials, together with sophisticated multivariate analysis methodologies, lays the groundwork for amplifying critical performance features. One of the most noteworthy benefits is a significant increase in reaction rate, which allows for quick and exact evaluation of target entities required for real-time monitoring or rapid diagnostics [172,173]. In the development of efficient electrochemical biosensors for point-of-care implementation, the coating on the biosensor surface must prevent the adhesion of foulants in complex biological samples. However, the evaluation of surface chemistry on the electrode surface for antifouling testing typically occurs using a specific medium and only one analyte, often within a highly controlled laboratory environment with simple sample solutions. Conversely, complex biological matrices comprise a significant number of extraneous molecules that have the potential to significantly impact sensitivity, particularly when the concentration of the target analyte is low. Thus, relying on a single biomarker for clinical decision-making is inadequate. Multiplexing in electrochemical biosensors represents a promising platform for enhanced disease detection [174].

Furthermore, the adoption of nanoarchitectural strategies significantly enhances detection limits, ushering in a new era of heightened sensitivity. Biosensors based on nanoarchitectonics can discern minimal quantities of analytes with unparalleled precision, leveraging the distinctive characteristics of nanomaterials. This breakthrough opens avenues for detecting biomolecules at levels that were previously imperceptible. The integration of nanostructures enhances surface area, facilitating the attachment of a high density of aptamers on the surface. This immobilization subsequently augments the rate of interactions and collisions between aptamers and their targets, leading to amplified signals within the detectable range [175].

Another critical aspect of nanoarchitectonics is enhanced stability, ensuring the longevity and reliability of electrochemical biosensors. Nanocomposites and nanohybrids improve structural resilience, mitigating the impact of environmental conditions and extending the operational lifespan of the biosensor. Functional groups in nanostructures enhance the electrode’s electroactivity, resulting in more stable bioreceptor immobilization on the electrode’s surface [4,176–178]. Using voltammetry, impedance, potentiometry and conductometry, researchers validated the detection limit of exosomes and other biological units in clinical samples [110]. Carbon nanostructures, metallic nanostructures (including metal-organic frameworks), transition metal dichalcogenides (TMDC) nanostructures, and DNA nanostructures are some of the most extensively employed nanomaterials in recent years. These compounds are used in aptamer or antibody-based electrochemical biosensors that detect exosomes. In compared to antibody-based electrochemical biosensors, the introduction of nanoarchitectonics in aptasensors has significantly improved selectivity, stability, and specificity.

### 4.1. Metal nanostructures

**Figure 2.**
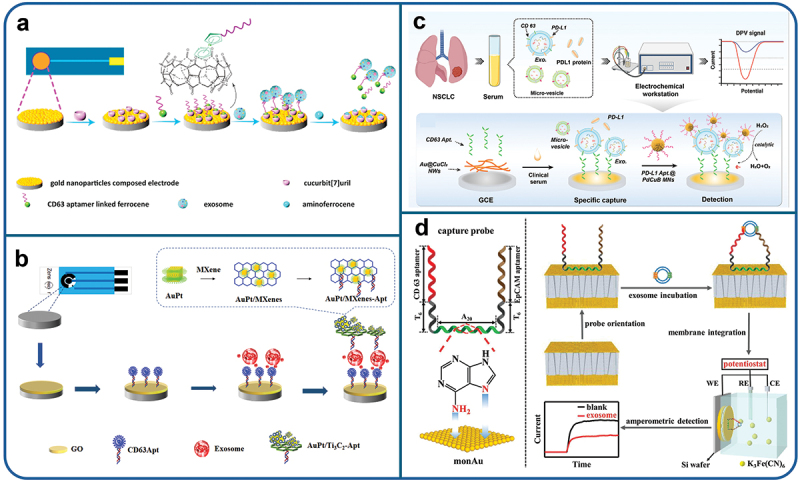
Schematic illustration of (a) Electrochemical aptasensor for exosomes capture and release based on specific host-guest interactions between cucurbit(7)uril and ferrocene. Reproduced by permission from [181], copyright [2011, Elsevier]. (b) An electrochemical immunosensor for the efficient detection of colorectal cancer-derived exosomes. Reproduced by permission from [182], copyright [2023, Micromachines]. (c) Sandwich electrochemical aptasensor for PD-L1+ exosome analysis. Reproduced by permission from [183], copyright [2023, Springer]. (d) Nanoporous alumina membrane based aptasensor for exosome detection reproduced from ref [184], copyright [2023, Elsevier].

Noble metal nanostructures including gold (Au), silver (Ag), platinum (Pt), and iridium (Ir) play a significant role in advancing electrochemical biosensors. Their surfaces can be easily modified, and the functionalization of metal nanostructures expands the active surface area, facilitating the entrapment of more target moieties and thus improving sensitivity [179]. Among these options, Au and Ag nanoparticles are commonly employed as the base of electrode interface for biosensor preparation due to their tremendous electrochemical, catalytic and electronic properties, coupled with high stability [180]. Liu et al. enhanced the Au NPs comprised electrode surface with cucurbit(7) uril by a self-assembly technique as demonstrated in Figure 2(a). Notably, the ferrocene (Fc) conjugated CD63 aptamers biomarker exhibited a remarkable detection limit down to 4.82 × 10^5^ exosomes/mL, demonstrating exceptional performance when applied to complex matrices such as plasma samples [181].

Fabrication of aptasensors suffers challenges of non-portability and slow diagnostic capabilities. To address this, a novel aptamer-based electrochemical portable biosensor incorporating cubic gold-platinum dendritic nanocrystals (AuPt DNs)/Ti_3_C_2_ for signal amplification was developed via a simple hydrothermal process. CD63 aptamer-attached graphene oxide was immobilized on a screen-printed carbon electrode (SPCE) used as a substrate to capture and detect exosomes, as depicted in Figure 2(b). The cubic structured AuPt DNs/Ti_3_C_2_-aptamer hybridized nanostructure exhibited efficient recognition of exosomes with a detection limit down to 20 particles/µL. This proposed electrochemical biosensor holds clinical value for early cancer detection [182]. Detection of PD-L1+ exosomes necessitates an extremely sensitive detection technique. Chang and his colleagues developed an electrochemical aptasensor based on Au@CuCl_2_ nanowires (NWs) and microporous nanospheres (MNs) composed of palladium, copper, boron alloys (PdCuB MNs) for the detection of PD-L1+ exosomes, as illustrated in Figure 2(c). The notable enzyme-mimicking catalytic feature of PdCuB MNs and enhanced conductivity of Au@CuCl2 NWs provided intense signals to the aptamer-based biosensor, enabling the detection of very low abundance of exosomes with a detection limit as low as 36 exosomes/mL. The electrochemical aptasensor operated successfully in complex matrices by overcoming biofouling and showing anti-interference by application of dual-target screening, proving to be a powerful tool for early diagnosis of Non-Small Cell Lung Cancer (NSCLC) even when applied to clinical samples with consistent results [183].

Another multiplex analysis was recently reported by Wang and his colleagues, and they introduceda sandwich-structured membrane engineered with GNPs for the detection of exosome [184]. This configuration involved assembling multilayer and monolayer GNPs on opposite ends of a nano porous alumina membrane as indicated in Figure 2(d). CD63 and EpCAM related specific probes sequences were immobilized on the monolayer gold nanoparticles, while the side of the multilayer gold nanoparticles was firmly adhered to a silicon wafer to mediate the rapid analysis of exosomes as shown in Figure 2(d). Exosomes binding during detection process led to a weakened flow of electric charge of Fe(CN)_6_^3-^, indicating amplified steric restraint and demonstrating quantitative exosomes analysis. The sandwiched configuration exhibited a detection limit of 2.8 × 10^2^ exosomes/µL. This innovative approach holds promise for extending to more sensitive, label free and fast quantification of various tumor-derived vesicles.

### 4.2. Transition metal dichalcogenides (TMDC) nanostructures

The two-dimensional (2D) nanostructures of TMDC, specifically MoS_2_, MoSe_2_, and MXenes, are utilised for their stability and to increase the sensitivity of biosensors. MoS_2_ is widely used in electrochemical biosensing because it has good stability, beneficial electrical properties (such as on/off ratio and extraordinary carrier mobility), and semiconductor properties that change depending on the layer. MoS_2_-incorporated field-effect transistors (FETs) are widely used for protein, DNA, and other biological molecule detection [185]. MXene-based nanomaterials have garnered a lot of attention lately from scientists because of their remarkable electrical conduction, enhanced chemical activity at the interface, and adjustable band structure. Because these TMDC nanomaterials have unique properties that enhance biosensor sensitivity, they are widely used on electrochemical interfaces. Furthermore, their two-dimensional arrangement makes it easier to modify metal nanostructures or create nanocomposites with other materials [186].

**Figure 3.**
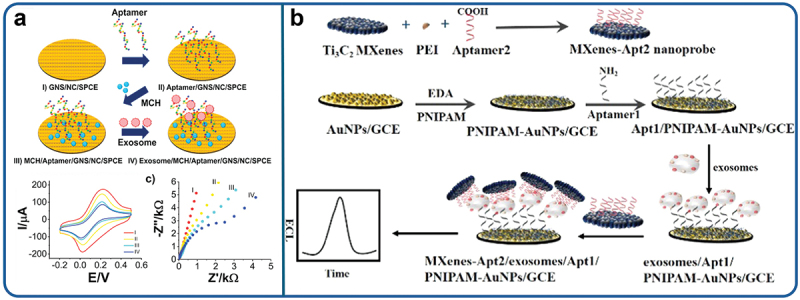
Illustration of ECL biosensor for exosomes detection by signal amplification strategy. (a) Construction of aptamer-2 modified Ti3C2 MXenes nanoprobes to improve the electron transfer on exosomes recognition. Reproduced by permission from [187], copyright [2020, Elsevier] (b) Fabrication of AuNPs modified GCE electrode with an activation of PNIPAM to immobilize aptamer-1 for capturing the target exosomes, and incubated with of MXenes-apt-2 nanoprobe and subjected to ECL characterization. Reproduced by permission from [188], copyright [2019, Elsevier].

MoS_2_ nanosheets (NS) were used to create an electrochemical biosensor with improved signal production by combining them with graphene nanoplatelets and chitosan in a nanocomposite as shown in Figure 3(a). When applied to the sensing interface, this nanocomposite demonstrated the complementary advantages of chitosan, graphene nanoplatelets, and MoS_2_ NS while offering an excellent surface on which to arrange the electrodeposition of metal nanoparticles. Moreover, highly conductive graphene nanosheets provided an increased surface area for the adherence of biorecognition aptamers [187]. The immobilisation of aptamers specific to epithelial cell adhesion molecules (EpCAMs) allowed for the capture of malignant exosomes from the entire sample volume, lowering the limit of detection (LOD) to 17 exosomes/µL.

Aptamer-adhered Ti_3_C_2_ MXenes NS were also employed for the electrochemiluminescence (ECL) biosensing of exosomes [188]. Utilizing the expanded surface area of Ti_3_C_2_ MXenes NS, an extensive amount of aptamers that are specific to the EpCAM protein were immobilized on the electrode surface in order to effectively detect exosomes as representef in Figure 3(b). Additionally, the excellent conductivity and catalytic properties of Ti_3_C_2_ MXenes NS increased the charge transfer on surface of electrode. As a result, the electrogenerated luminol chemiluminescence signals were amplified without using co-reactors like H_2_O_2_. Consequently, the reported findings indicate the successful detection of MCF-7 exosomes with a remarkably low detection limit of 125 exosomes/µL – more than 100 times lower than that achieved by ELISA.

### 4.3. Organic framework nanostructures

Metal organic frameworks (MOFs) and covalent organic frameworks (COFs) being two renowned skeletal nanomaterials, are novel crystalline porous structures generated by the self-assembly of organic ligands and inorganic metal nodes. These nanostructures are highly suitable for enhanced biosensing because of flexible porosity, increased specific surface site density, chemical tunability, and robust sensing properties [189]. The redox activity mediated by the active metal ions within MOFs serves as a signal probe in electrochemical bio-sensing, allowing the capture of amplified signals for biosensors and precise detection of analytes with high sensitivity. These skeletal frameworks can also enclose nanoparticles as carriers to enhance the catalytic properties of composite materials [190].

**Figure 4.**
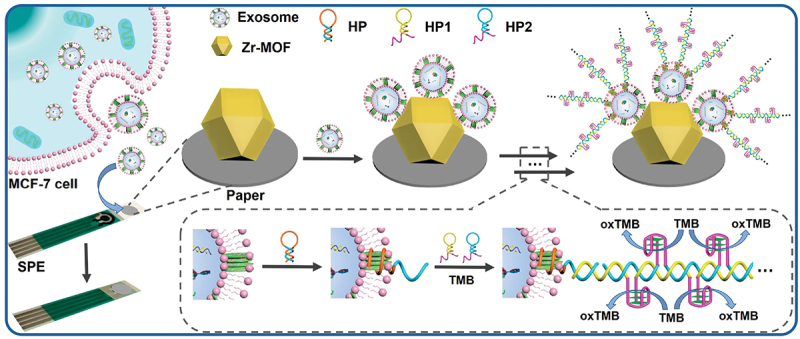
Schematic illustration of the mechanism of the paper-based electrochemical biosensor built on Zr-MOFs for cancerous exosome assay. Reproduced by permission from [191], copyright [2021, American Chemical Society].

Zr-based MOFs have emerged as key components in the detection of exosomes, considerably enhancing signals in electrochemical biosensors. Liu’s group developed a sensitive and portable paper built point-of-care biosensor. The biosensor is principally based on an aptamer-based detecting mechanism, with Zr-MOFs initiating the hybridization-chain reaction (HCR) for signal amplification via DNA-zyme synthesis as illustrated in Figure 4. The Zr-MOF-modified paper-based biosensor demonstrated very sensitive exosome analysis with a detection limit as low as 5 × 10^3^ mL^−1^ by leveraging the improved signal output of HCR and the effective catalytic action of DNA-zyme. This biosensor stands out for its low cost, ease of use, and applicability for resource-constrained environments [191]. The interaction of Zr^4+^ ions in the Zr-MOF-based multi-layered aptasensors not only occurs with exosomes but also with aptamers, leading to false positives and significant background noise. To address this issue, Li and his colleagues introduced an aptasensor decorated with Pd nanoparticles and hemin-enclosed UiO-66 MOFs as markers for signal amplification. This approach aimed to eliminate false positive responses and reduce background noise. CD63-specific aptamers were immobilized on the surface of magnetic Fe_3_O_4_ particles coated with polydopamine (PDA), along with UiO-66-NH_2_ for exosome capture. As a result, the prepared aptasensors exhibited enhanced sensitivity with a detection limit of 86.2 particles/µL and demonstrated high selectivity towards exosomes originating from various sources, including the MCF-7 cell line and HeLa cell line [192].

### 4.4. Carbon nanostructures

Over the past decade, carbon-based nanostructures such as graphene, fullerenes, carbon nanotubes or carbon dots possess significant potential to meet the requirements of electrochemical biosensors [193]. Among them, graphene and its derivatives, graphene oxide (GO) and reduced graphene oxide (rGO) have been widely employed in electrochemical biosensing due to their remarkable electrical and mechanical properties. Their large surface-to-volume ratio, enriched oxygen content and efficient formation of nanocomposites with metal nanoparticles, metal oxides or polymers, make them advantageous in electrochemical sensing approaches [194].

**Figure 5.**
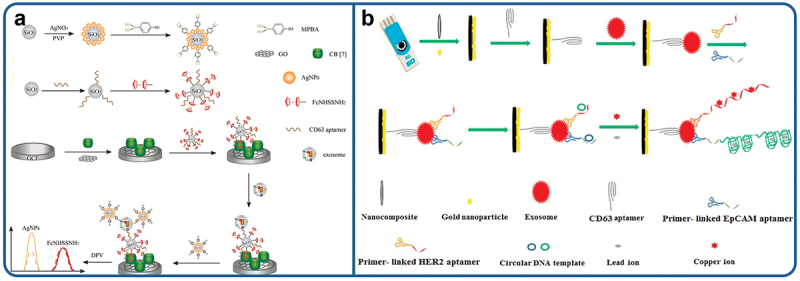
Schematic illustration of (a) Ratiometric electrochemical sensor for the analysis of exosomal glycoproteins. Reproduced by permission from [195], copyright [2021, Elsevier]. (b) The proposed electrochemical aptasensor for the multiplex detection of exosome biomarkers. Reproduced by permission from [196], copyright [2022, Elsevier].

A ratiometric electrochemical biosensor based on GO-cucurbit modified electrode was reported [195]. To identify glycoproteins on exosomes, silica-silver (SiO_2_@Ag) core-shell nanoparticles were incorporated with mercaptophenyl boronic acid (MPBA) and CD63 specific aptamer as a recognition element. The glassy calomel electrode modified with GO-cucurbit immobilizes CD63-specific aptamer-SiO_2_@-ferrocene carboxamide (FcNHSSNH_2_) for the selective capture of exosomes via host-guest interaction. Simultaneously, the MBPA-SiO_2_@Ag tracer attaches to exosomes as MPBA selectively hybridizes cis-diol framework of glycoprotein on the exosomal surface. The abundant coating of silver nanoparticles on the SiO_2_ surface to the amplification of electrochemical signal. The charge transfer ratio of Ag nanoparticles and FcNHSSNH_2_ was demonstrated as an experimental finding as shown in Figure 5(a) that enhanced the precision and sensitivity of the reported biosensor. The biosensor showed the detection limit to be 368 exosomes/µL and can be applied to recognize glycoproteins on exosomes in humans blood serum samples, indicating a promising future for clinical applications. Moreover, an extremely sensitive multiplex electrochemical aptasensor was developed by Hashkavayi and her colleagues for screening HER-2 and EpCAM positive exosomes. The electrode surface was modified using the composite consisting of amine-group modified multi-wall carbon nanotube (MWCNT), chitosan and ionic liquid as shown in Figure 5(b). This nanocomposite facilitated the effective electrodeposition of Au NPs and the immobilization of aptamers on a screen-printed carbon electrode (SPCE). Modification of the electrode surface, combined with rolling circle amplification (RCA), significantly amplified the electrochemical signals and greatly enhanced the sensitivity of the electrochemical aptasensor. The detection limit was recorded to be 1 exosome/mL [196].

### 4.5. DNA nanostructures

**Figure 6.**
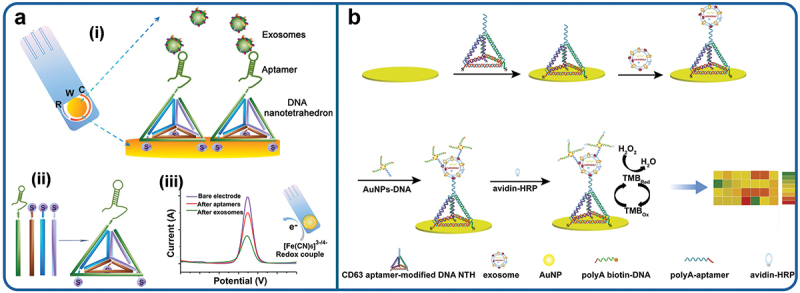
Schematic illustration of the (a) NTH-assisted electrochemical aptasensor. (i) Aptamer-containing NTHs were immobilized via three thiol groups onto the gold electrodes for direct capture of exosomes in suspension. (ii) Facile self-assembly of DNA nano tetrahedral. (iii) Redox signal changes after aptamer immobilization and after incubation with exosomes. Reproduced by permission from [197], copyright [2017, American Chemical Society] (b) Electrochemical aptasensor for exosomal proteins profiling based on the DNA nano tetrahedrons. Reproduced by permission from [198], copyright [2020, Elsevier].

DNA nanotechnology has found widespread application in biosensors for its signal amplification, predictiveness and biocompatibility. DNA Nano tetrahedrons are the DNA nanostructures formed by the self-assembly of the four distinct single stranded DNA sequences that are oriented specifically and possess resilient mechanical framework. These features secure the specific position and density of aptamers on the surface of electrodes offering solutions to the challenges like aptamer aggregation, entanglement and spatial hindrance in conventional electrochemical biosensors [179]. Wang et al. described a nano tetrahedron (NTH)-enhanced aptasensor for hepatocellular exosome detection [197]. As shown in Figure 6(a), this novel approach combines DNA-built nanostructures, aptamer technology, and electrochemical techniques to quickly isolate and identify exosomes. Aptamer tetrahedral immobilization considerably improves their accessibility to artificial nucleobases in exosome suspension. The Nano Tetrahedron-modified aptasensor detected exosomes 100 times more sensitively than aptasensors modified with single-stranded aptamers, with a detection limit as low as 2.09 × 10^4^ /mL. This method laid the foundation for quantifying exosomes in complex biological fluids. Additionally, Jiang et al. integrated Au NPs and enzymes signal amplification to fabricate an aptamer-enhanced DNA NTH electrochemical biosensor to detect HepG2- -generated exosomes as depicted in Figure 6(b). The reported limit of detection (LOD) reported was as low as 1.66 × 10^4^ exosomes/mL [198].

Recently, DNA walkers, a substantial branch of robust DNA nanotechnology, have been employed for the amplified and unidirectional transmission of detection signals. Miao et al. introduced CD63 aptamers and utilized the convenient segregation of magnetic Fe_3_O_4_@Au NPs for the specific detection of exosomes. Their electrochemical aptasensor was built on multi-legged DNA based walker to recognise cervical cancer cells released exosomes. These DNA walkers merge the benefits of cyclic DNA amplification and Electrochemical screening, which can significantly enhance exosome detection [199].

## 5. Limitations and future perspectives

The article provides a concise overview of exosome properties, their significance in diseases, and recent advancements in utilizing aptamer-based nanoarchitectonics for robust biosensors with enhanced efficiency. Recent advances in nanomaterials have played a pivotal role in biosensor development, offering the potential to create highly sensitive, targeted, and label-free biosensors [185]. The combination of nanomaterials with high-affinity aptamers broadens and improves biosensor sensitivity techniques [200]. This approach leverages the advantage of the exceptional electrochemical properties of nanomaterials such as gold nanoparticles, MOFs, graphene, and other 2D nanomaterials [186]. The interaction between nanomaterials and aptamers enhances the performance of biosensors, creating a diverse platform for exosome detection applications [201]. However, several challenges need to be addressed to enhance the performance and practicality of these biosensors.

First, the success of aptamer-based nanoarchitectonics relies on the meticulous selection of aptamers characterized by high specificity and affinity for target exosomes. The existing challenges in aptamer selection and exosome isolation techniques underscore the need for intensified research efforts, as several critical technical issues and obstacles still require further refinement. The highly heterogeneous nature of exosomes, their nanometric size, and complex chemical and physical characteristics in body fluids necessitate significant improvements in specific separation-capture performance. Current isolation techniques for exosomes exhibit defects and variations in experimental reagents, along with procedural complications, leading to some degree of damage to exosome purity and separation. Aptasensors, although promising, still face limitations in comprehensively analyzing exosomes and their cargo through a single-step isolation-trap-recognition process. To ensure repeatability and consistency in exosome detection using aptasensors, the use of a standard calibrated sample is crucial [119]. Aptasenising, like other analytical techniques is very sensitive to sample handling during exosome detection. Standardizing the detection procedure would reduce experimental inconsistencies and ensure reproducible and reliable results. Therefore, further in-depth research and knowledge are required for targeted detection and sensing techniques for exosomes. While over 10 commonly found proteins with different functional groups have been identified in exosomes so far, relying solely on these proteins may not effectively distinguish exosomes from various sources [118]. Hence, there is a need for the identification of new exosomal biomarkers and their confirmation for specific cancers. Additionally, aptamers targeting complete exosomes, peptides, and myelin are necessary, rather than focusing solely on proteins [119]. In various diseases, exosomes specific to the cells possess their unique molecular fingerprints, requiring aptasensors to have sharp resolution for exosomal targets. However, the limited number of aptamers for exosomes necessitates studies to refine aptamer selection methods, particularly emphasizing intact exosomes to optimize their identification. A comprehensive understanding of the universality and specificity laws governing aptamer selection has emerged as a critical factor influencing the development of aptamers with heightened specificity for exosomes [117].

Furthermore, efforts to enhance exosome identification specificity include integrating marker assays, offering a promising approach to improve accuracy and discern-secreted exosomes from diverse sources. Despite these advancements, achieving a notably high level of specificity in exosome detection remains a multifaceted challenge, necessitating ongoing exploration and innovation [119]. Second, despite achieving sensitivity to picomolar levels in laboratory experiments, the transition to real samples introduces challenges stemming from sample complexity. The presence of biological matrices, such as serum or plasma, poses a risk to the accuracy, stability, and specificity of the biosensor. Notably, different subtypes of extracellular vesicles share common tetraspanin proteins, that reduces the specificity rate of exosomes [202]. To address this issue, concurrent applications of diverse aptamers targeting a group of tetraspanin proteins can enhance precision and specificity. Future studies will prioritize overcoming interference from complex matrices to ensure accurate and reliable results. The integration of microfluidic chips in the development process can efficiently separate complex matrices, improving the biosensor’s performance. Additionally, the instability of chemical modifications within nanomaterial-functionalized biosensors, such as those involving amino, thiol, aptamer, or antibody groups, hinders prolonged storage. Therefore, efforts should be directed toward optimizing the storage conditions for these chemically modified groups through repeated experiments. Finally, the variations in quantification units for exosomes across studies pose a challenge in making meaningful comparisons, particularly concerning the limits of detection [203]. To address this issue, it is crucial to establish a standardized and unified unit for the quantification of exosomes, providing a common ground for accurate comparisons. Additionally, there is a growing trend in utilizing nanomaterials to advance the development of programmable, versatile, and adaptive intelligent structures. By integrating nanomaterial structures with digital simulations, the creation of intelligent nanostructures becomes feasible. This innovative approach enhances the predictability of characteristics and functions, offering a promising avenue to expedite and advance research in the field of exosomes [204]. Despite of several issues, like non-specific adsorptions, inadequate storage conditions and heterogeneity in samples nanoarchitectonics advanced aptamer-based electrochemical biosensors have reported promising results for clinical applications. We, therefore, believe that aptamer-based electrochemical biosensors with nanoarchitectonics incorporation will provide precise analysis of exosomes and their molecular cargos ultimately enabling non-invasive disease monitoring and contributing to improvement in the survival rate and the quality of life for the patients.
